# Energetics and Ecological Implications of Bacterial Electron Transport Chains

**DOI:** 10.1111/1758-2229.70338

**Published:** 2026-04-06

**Authors:** Ningdong Xie, Allison M. Brown, Xin Wang

**Affiliations:** ^1^ Department of Microbiology and Cell Science, Institute of Food and Agricultural Sciences University of Florida Gainesville Florida USA; ^2^ Department of Microbiology, Genetics, and Immunology Michigan State University East Lansing Michigan USA

## Abstract

Energy conservation by the electron transport chain (ETC) is a fundamental cellular process. Utilising membrane‐bound protein complexes, the ETC allows cells to conserve chemical energy into proton motive force (PMF), which further drives the production of adenosine triphosphate (ATP) used for cell chemistry. In bacteria, diverse respiratory chain compositions exist, enabling them to adapt and thrive in a variety of habitats. In this mini‐review, we discuss the energetic efficiency of bacterial respiratory chains to reveal underlying design principles and further explore its implications in supporting survival of bacteria utilising different energy sources. Particularly, our cross‐database analyses suggest that the incorporation of cytochrome *bc*_1_ complexes in ETCs, which enables efficient PMF generation, is associated with the dominant bacterial taxa in global oceans and soil, thus highlighting the ecological significance of ETC energetic efficiency.

## Graphical abstract

This review synthesises vast knowledge on bacterial electron transport chains with an emphasis on the energetics and underlying design principles. It further explores the varied respiratory chain compositions across different bacterial species using diverse energy sources to reveal potential implications of energy metabolism in supporting bacterial survival in diverse environments.

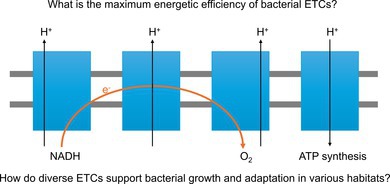

## 1 Introduction

Energy conservation by the electron transport chain (ETC) is a fundamental process in life. The ETC employs membrane‐bound protein complexes to convert chemical energy in reduced molecules into the proton motive force (PMF), an electrochemical gradient across lipid membranes. A typical ETC consists of five protein complexes termed NADH dehydrogenase (NDH; Complex I), succinate dehydrogenase (SDH; Complex II), cytochrome *c* reductase (Complex III), cytochrome *c* oxidase (IV), and adenosine triphosphate (ATP) synthase (Complex V). Electrons from NADH or succinate are first passed to quinones through Complex I and II, respectively, to enter the ETC. Quinols (reduced form of quinone) further donate electrons to Complex III, eventually passing electrons to another mobile electron carrier, cytochrome *c*. The soluble cytochrome *c* passes electrons one at a time to a terminal electron acceptor through Complex IV, completing the redox reaction. Compared to the mitochondrial ETC, bacterial ETCs are diverse in their compositions and energetics, providing the flexibility to access energy from various sources.

The generation of ATP by ETC relies on the proton‐pumping mechanisms of the various ETC components to establish the PMF. Once a sufficient gradient is formed, protons will travel down the electrochemical gradient back into the cells through the ATP synthase, which operates via a rotational mechanism and catalyses the conversion of adenosine diphosphate (ADP) to ATP. It costs an average of 3–4 protons to generate one molecule of ATP by ATP synthase (Yoshida et al. 2001). Although the scheme of the electron transport chain has been discussed extensively in the literature, its architectural principle and energetic implications remain less appreciated. In this mini review, we focus on the energetics and diversity of bacterial ETCs to reflect the architectural principles and implications of this fundamental cellular process.

## 2 Architecture of Bacterial Electron Transport Chains

**FIGURE 1:**
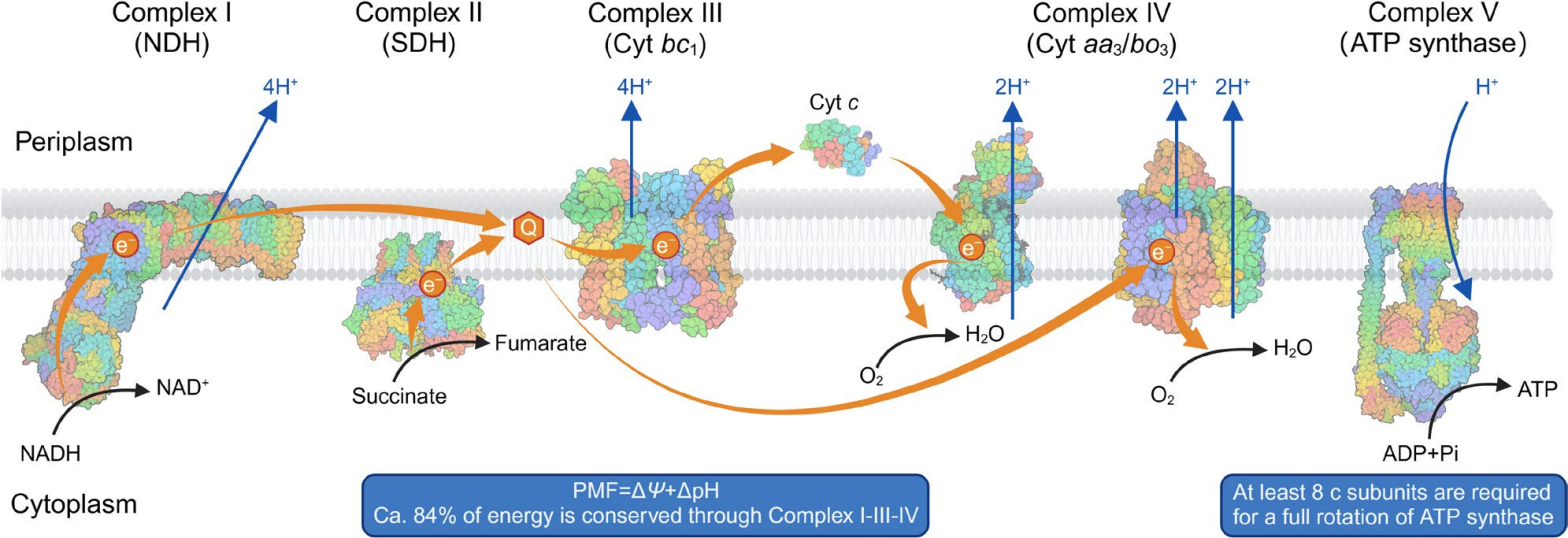
Diagram of bacterial electron transport chains. Orange arrows indicate typical pathways of electron transport during bacterial respiration, where the electrons are donated from NADH/succinate to quinone (Q) and further to the terminal electron acceptor (e.g., O_2_) either directly or indirectly via the cytochrome (Cyt) *bc*_1_ complex. Blue arrows indicate proton pumping from cytoplasm to periplasm to generate proton motive force (proton numbers corresponding to oxidation of one NADH and transfer of two electrons) and diffusing back to drive ATP synthesis. Representative structures of Complexes I‐V and cytochrome (Cyt) *c* are retrieved from RCSB Protein Data Bank (PDB), with NADH dehydrogenase (NDH, Complex I, PDB ID: 7P61), succinate dehydrogenase (SDH, Complex II, 2ACZ), cytochrome *bo*_3_ (Complex IV right, 7CUB), and ATP synthase (Complex V, 6OQR) derived from *Escherichia coli* while cytochrome *bc*_1_ (Complex III, 2YIU), cytochrome *c* (3 M97), and cytochrome *aa*_3_ (Complex IV left, 3HB3) derived from *Paracoccus denitrificans* . The diagram is created with BioRender (agreement number: WP26U0WCIG).

Energy conservation by ETCs follows a simple yet elegant design principle. The ETC complexes in cell membranes use redox energy to establish an electrochemical gradient. With this simple architecture, different molecules converge their chemical energy into PMF through various entry points to the ETC. The principal reactions catalysed by ETC are quinol oxidation and reduction of terminal electron acceptors. There are mainly two sets of ETC structures that can complete this process, either with or without the participation of the cytochrome *bc*_1_ complex (Figure 1). When the cytochrome *bc*_1_ complex is absent in some respiratory chains, as in *Escherichia coli* , reduced quinols transfer electrons directly to the terminal oxidase (Borisov, Murali, et al. 2011). Depending on whether oxygen and other chemicals such as nitrate or sulphate are used as the terminal electron acceptor, this process is termed aerobic and anaerobic respiration, respectively.

### 2.1 ETC Entry Points for NADH, Succinate, and Other Substrates

A main entry point of ETC is through universal energy carriers such as NAD(P)H. These electron carriers are produced as byproducts of central metabolism such as glycolysis and the citric acid cycle and are fed into the ETC to establish the electrochemical gradient. NADH dehydrogenase (NADH‐quinone oxidoreductase; Complex I) is an enzyme that facilitates NADH oxidation and transfers electrons to the next module of the respiratory chain, which are typically quinone molecules (Yagi 1991). This redox energy can be partially conserved as PMF using the proton‐pumping NDH‐1 complex or completely lost as heat if the alternative NDH‐2 complex conducts the redox reaction (Yagi et al. 1993; Heikal et al. 2014). In many pathogenic bacteria, a sodium‐pumping NADH dehydrogenase also exists to provide access to the respiratory chain (Reyes‐Prieto et al. 2014).

Succinate dehydrogenase (SDH; Complex II) is an enzyme participating in the citric acid cycle to catalyse the conversion of succinate to fumarate, in which the oxidised flavin adenine dinucleotide (FAD^+^) is used as the electron acceptor to generate FADH_2_ (Hederstedt and Rutberg 1981). FADH_2_ further donates its electrons to quinone through a series of iron–sulphur clusters in the SDHB subunit (Cecchini 2003; Moosavi et al. 2019). Scientific literature and textbooks often mischaracterise Complex II as the ETC entry point for FADH_2_ rather than succinate (Gnaiger 2024). In fact, the converging point for different substrates to access the respiratory chain is more at the quinone pool, where a variety of dehydrogenase enzymes can gain access by oxidising these substrates and reducing quinones (Wallace and Young 1977; van Beilen and Hellingwerf 2016).

Beyond NADH and succinate, an expansive array of substrates, including sulphide, dihydroorotate, proline, alanine, glycerol‐3‐phosphate, and various organic acids and alcohols, can enter bacterial ETCs via diverse quinone reductases and alternative pathways. This metabolic plasticity facilitates energy acquisition and survival across a wide range of bacterial taxa, enabling them to occupy diverse ecological niches and endure fluctuating environmental conditions (Hedrich et al. 2011; Marreiros et al. 2016; Bryce et al. 2018; Jones and Santini 2023; Chen et al. 2025).

### 2.2 Completing Electron Transfer to Terminal Electron Acceptors

In bacteria, Complex IV alone or together with Complex III contributes to energy conservation by oxidising quinol and reducing terminal electron acceptors. Compared to ETC compositions in mitochondria, a main difference of bacterial ETCs is that some lack the cytochrome *bc*_1_ complex (Complex III). Because of this, bacterial terminal oxidases (Complex IV) can be quinol oxidases or cytochrome *c* oxidases, i.e., accepting electrons from quinol either directly or indirectly through cytochrome *bc*_1_ complex (Figure 1).

Bacterial terminal oxidases (oxygen reductases) consist of mainly three types, depending on the prosthetic groups within, i.e., heme‐copper, non‐heme di‐iron carboxylate, and heme *b/d* (Borisov, Gennis, et al. 2011). Among these, only the heme‐copper oxygen reductases contain both quinol and cytochrome *c* oxidases with the other two classes consisting of only quinol oxidases. Cytochrome *c* oxidase represents the mitochondrial terminal oxidase cytochrome *aa*_3_. Phylogenetic research has suggested that this enzyme originated in proteobacterial chemolithotrophs, most likely ancestors to extant iron‐oxidisers like *Acidithiobacillus ferrooxidans* . From there, the primitive cytochrome *c* oxidase would spread to other prokaryotes via lateral gene transfer and develop into the modern terminal oxidases (Esposti 2020).

Cytochrome *bc*_1_ complexes vary greatly among organisms in the numbers of protein subunits, with some species such as *Paracoccus denitrificans* maintaining only essential subunits needed for electron transfer, whereas others have more accessory protein subunits (Xia et al. 2013). The essential subunits present in all *bc*_1_ complexes are the subunits containing cytochrome *b* hemes, a cytochrome *c*_1_ heme, and the iron sulphur cluster (Rieske centre). A unique feature of the cytochrome *bc*_1_ complex is that it employs the ‘Q‐cycle’ mechanism to conserve energy into PMF (Mitchell 1961, 1975). This process, conferring a greater PMF to organisms that incorporate this complex by translocating four protons per quinol molecule rather than two, has been described thoroughly in past review articles or textbooks (Trumpower 1990; Cramer et al. 2011).

Interestingly, cyanobacteria and other phototrophs use a related complex called cytochrome *b*_6_ *f* for electron transfer in both respiration and photosynthesis light reactions (Lea‐Smith et al. 2016). This complex shares a common core with the *bc*_1_ complex, consisting of a Rieske iron–sulphur centre, a *b*‐type heme, and a *f‐*type heme (Kurisu et al. 2003), but also differs from the *bc*_1_ complex in the presence of an additional *c*‐type heme in the negative side of the membrane (Baniulis et al. 2008). Due to the highly related structures of these two complexes, it is widely thought that the two emerged from the same evolutionary origin. However, the exact nature of this evolution is still under debate. It has been found that no significant lateral gene transfer contributed to the emergence of the *bc*_1_ complexes, and therefore some believe that a primitive *bc*_1_ complex must have been present in the last universal common ancestor (LUCA), before the separation of the three domains of life (Schütz et al. 2000). It has also been suggested that *bc*_1_ complexes first emerged in some early bacterial ancestors and later transferred to archaea by lateral gene transfer. Genetic and structural analyses of the two complexes have revealed that the cytochrome *b* polypeptide of the *bc*_1_ complex corresponds to two separate polypeptides in *b*_6_ *f*: the cytochrome *b*_6_ and subunit IV. Two scenarios have therefore been proposed regarding the ancestral *bc*_1_ complex: the first *bc*_1_ complex may have resembled a *bc*_1_ complex, containing a ‘long’ cytochrome *b* that later was split into the subunit IV and cytochrome *b*_6_ found in photosynthetic organisms. Alternatively, an ancestral *b*_6_ *f*‐like complex may have fused cytochrome *b*_6_ and subunit IV, resulting in a long cytochrome *b*. While it is still unclear which of these scenarios, if either, is correct, phylogenetic evidence has led some researchers to believe the fusion scenario is more likely. After the emergence of the ancestral *bc*_1_ complex, evolution to modern complexes would have been driven by the increasing availability of high‐reduction‐potential electron acceptors like oxygen (Dibrova et al. 2013).

In anaerobic ETCs, compounds such as nitrate, nitrite, nitric oxide, dimethyl sulphoxide (DMSO), polysulphide, tetrathionate, thiosulphate, sulphur, ferric iron, and manganese oxides serve as terminal electron acceptors. These processes utilise a diverse array of electron carriers and terminal oxidoreductases to facilitate energy conservation (Gescher and Kappler 2013; Marreiros et al. 2016; Jones and Santini 2023; Chen et al. 2025). Such metabolic modularity enables specific bacterial taxa to maintain growth and survive within hypoxic or strictly anaerobic environments.

### 2.3 ATP Production

ATP synthase (Complex V) are ubiquitous among respiring organisms. They couple proton translocation with ATP synthesis or hydrolysis. Most bacteria express F‐ATPase consisting of an F_1_ and F_0_ region. The F_1_ region, located in the cytosol and anchored by F_0_, consists of an α_3_β_3_ hexamer along with the γ, δ, and ε subunits. F_0_ is a membrane‐embedded region formed from the *ab*_2_ *c*_8–17_ subunits (Walker 2013; Nakanishi‐Matsui et al. 2016; Guo et al. 2019). This variation in the number of *c* subunits, which is seen between different organisms, contributes to the proton/ATP stoichiometry of ATP synthase. It is generally accepted that one full rotation in ATP synthase results in 3 ATP molecules (Ferguson 2000; Yoshida et al. 2001). The number of *c* subunits in different bacteria are likely evolutionarily selected to best meet cell needs for both energy conservation and environmental adaptation. For example, the number of *c* subunits in cyanobacteria and other oxygenic phototrophs are among the largest found in nature. It has been suggested that this helps maintain a proper level of PMF for ATP production and avoid excessive membrane potential that could lead to photodamage (Davis and Kramer 2020).

## 3 Energetics Across the ETC Branch Points

The proton pumping capacity of ETC membrane complexes determines the energy conservation efficiency. One intriguing question to ask is thus about the efficiency of bacterial ETCs, including both energy conservation efficiency by ETC and energy conversion efficiency by ATP synthase. Together with distribution of energy efficiency profiles among different bacteria, it might offer clues on the contribution of energy efficiency to bacterial survival in different habitats.

The architectural diversity of bacterial ETCs creates a range of energy conservation efficiencies that can be systematically analysed as a series of modular branching points. We first analyse ETC with a complete set of proton‐pumping protein complexes as an example. For a typical NADH dehydrogenase, four protons are pumped to the positive side of the membrane for every 2 e^−^ extracted from NADH. Succinate dehydrogenase can facilitate the electron transfer from succinate to quinones but does not move any protons across the membrane. The reduced quinol molecules further donate electrons to the cytochrome *bc*_1_ complex and release four protons to the positive side of the membrane through the Q‐cycle (Mitchell 1975). The soluble cytochrome *c* released from the cytochrome *bc*_1_ complex donates electrons one at a time to cytochrome *aa*_3_, pumping two additional protons to the positive side of the membrane (Figure 1). The ETC with a complete set of protein complexes thus has the electrogenic ratio of 5 H^+^/e^−^ by extracting energy from NADH, or 3 H^+^/e^−^ from succinate.

We can determine the ETC energetics using NADH oxidation as an example. The reduction potential difference (Δ*E*) between NAD^+^/NADH (*E*_m_ = −0.32 V) and O_2_/H_2_O (*E*_m_ = 0.82 V) has a span of 1.14 V (Nicholls and Ferguson 1992). Using the equation △G=−nF△E where *n* is the number of electrons and *F* being the Faraday constant (96,485 C mol^−1^), the complete oxidation of NADH releases ca. 220 kJ/mol energy under the standard conditions (Feiner and McEvoy 1994). During respiration, this redox energy is partly conserved as PMF with the rest released as heat to drive the reaction forward. The PMF consists of two components, i.e., the electrical potential (Δ*ψ*) and the proton gradient (ΔpH). The PMF (∆*p*) is thus represented by the equation ∆*p* = Δ*ψ* + RT/*F* ln([H^+^]_P_/[H^+^]_N_) where *R* is the gas constant (8.314 J mol^−1^ Kelvin^−1^), *T* is the absolute temperature (Kelvin), *F* is the Faraday constant (96,485 C mol^−1^), and [H^+^] represents proton concentrations of the positive and negative sides of the membrane (Mazat et al. 2013). Under a typical bacterial membrane potential of ca. −0.15 V (Tran and Unden 1998; Lo et al. 2007), ΔpH of 0.7 (Maurer et al. 2005), and the temperature of 25°C, proton translocation across the bacterial cell membrane conserves ca. 18.5 kJ/mol energy, with the majority contributing from Δ*Ψ*.

The net energy conservation efficiency of the ETC branch during aerobic respiration is thus ca. 84% by translocating 10 H^+^ across the membrane per NADH (Figure 1). Therefore, bacteria employing a complete I‐III‐IV architectural branch have nearly maximised their energy conservation efficiency. It should be noted that the maximum energy available from NADH oxidation can be slightly increased by altering the concentration ratios of NAD^+^/NADH and O_2_/H_2_O in the cellular environment, thus potentially lowering the conversion efficiency. However, modularity allows for alternative branches that deviate from this maximum. For instance, replacing NDH‐1 with the non‐pumping NDH‐2 eliminates the initial 4 H^+^/2e^−^ capture, reducing the total efficiency of the NADH to oxygen path to approximately 50%. Similarly, bypassing the *bc*_1_ complex via direct quinol oxidases, such as cytochrome *bo*_3_, removes the four‐proton yield of the Q‐cycle, dropping the conservation efficiency of the NDH‐1 initiated chain to approximately 67%. For alternative substrates like succinate, the redox span to oxygen is smaller (∆*E* = 0.79 V) (Berg et al. 2015), yielding ca. 152 kJ/mol; depending on whether the *bc*_1_ branch is utilised, the efficiency of this specific path typically ranges from 49% to 73%.

Bacteria using alternative energy sources and terminal electron acceptors have even reduced free energy (Hedrich et al. 2011; Berg et al. 2015). Substrates such as sulphide, hydrogen, formate, thiosulphate, and ferrous iron enter the ETC chain through specialised reductases, each representing a distinct ∆*E* (Thauer et al. 1977). For example, the oxidation of ferrous ions (Fe^2+^/Fe^3+^, *E*_m_ of *+*0.77 V at pH 2) or sulphide (H_2_S/S^0^, *E*_m_ of ≈ +0.025 V or slightly higher at pH 2) provides a much narrower redox gap than NADH. In anaerobic respiration, the available free energy is further constrained by the selection of terminal acceptors such as nitrate, nitrite, nitric oxide, DMSO, or sulphur. For example, the oxidation of succinate (*E*_m_ of +0.03 V) coupled with the reduction of nitrate (*E*_m_ of +0.43 V) yields an energy span significantly smaller than aerobic NADH oxidation. In these low‐voltage anaerobic branches, incorporating the cytochrome *bc*_1_ complex can be advantageous to maximise the proton translocating capacity and ensure that even marginal redox spans are effectively conserved as PMF. By analysing these configurations as discrete branches, it becomes clear that while I‐III‐IV architecture provides a theoretical ceiling, alternative branching points allow bacteria to balance high‐efficiency energy conservation with the metabolic and environmental demands of their specific ecological niches.

The energy conversion efficiency by ATP synthase introduces a final critical constraint within the respiratory architecture. This conversion efficiency is primarily governed by the stoichiometry of the *c*‐ring of the F_0_ region. For proton‐driven ATP synthases, the number of *c* subunits determines how many protons are required to drive a full rotation to synthesise 3 ATPs (Vik and Antonio 1994). The maximum efficiency of ATP synthase, i.e., using the minimum number of *c* subunits to drive a full rotation of ATP synthase, can thus be easily calculated. We use *E* . *coli* as an example, in which an estimate of 47 kJ/mol energy is required for ATP synthesis under different respiration conditions (Tran and Unden 1998). Since proton conservation requires about 18.5 kJ/mol energy under typical cellular conditions, it would need at least 7.6 *c* subunits (47 × 3/18.5) to drive a full rotation of ATP synthase (Figure 1). It is thus not surprising that the smallest number of c subunits found in nature is 8 (Walker 2013; Davis and Kramer 2020). Under most conditions, it is likely safe to state that evolution has maximised the design spaces for both energy conservation by ETC and conversion by ATP synthase.

## 4 ETC Compositions in Bacteria Using Different Energy Sources

Energy conservation efficiency has a direct contribution to fluxes according to the flux‐force relationship $△G=-\mathrm{RT}\;\ln\left(J^{+}/J^{-}\right)$, in which *R* is the gas constant, *T* is the temperature in kelvin, and $J^{+}$ and $J^{-}$ represent the forward and reverse flux, respectively (Beard and Qian 2007). With higher energy conservation efficiency (i.e., lower energy loss), there is a reduced pathway flux. Therefore, bacteria with higher‐efficiency ETCs potentially generate smaller energy fluxes compared to those with lower efficiency, which could have a large impact on dynamics of ATP‐consuming chemistry. High energy conservation efficiency with low pathway flux has been proposed to support evolution of multicellular structures and could also be related to symbiosis of bacterial populations. In oligotrophic environments that only allow low growth rates, an efficient energy conservation can play a key role in success of a population's survival. In contrast, low energy conservation efficiency with high pathway flux implies a fast consumption of resources, potentially leading to a dominant population in case plentiful resources are available (Neijssel and de Mattos 1994). To support this notion, we can seek some clues by looking into energy utilisation efficiencies in bacteria using different energy sources (Table 1).

**TABLE 1:** Energetic efficiencies and implications of bacteria using different energy sources.

Trophic types	Chemoorganotrophs	Chemolithotrophs	Phototrophs
Model bacteria for ETC studies	*Escherichia coli* *Corynebacterium glutamicum*	*Paracoccus denitrificans* *Nitrosomonas europaea* *Kuenenia stuttgartiensis*	Cyanobacteria
Energy sources	Organic compounds	Inorganic/C_1_ compounds	Light (photosynthesis) Intracellular storage C (respiration)
Electron donors into ETCs	NADH, succinate, lactate, and malate, etc.	Inorganic/C_1_ compounds and NADH	Water (photosynthesis) NAD(P)H and succinate (respiration)
Typical PMF generation	1~4 H^+^/e^−^	Up to 5 H^+^/e^−^	3 H^+^/e^−^ (photosynthesis) 1~5 H^+^/e^−^ (respiration)
Typical energetic strategies	Low energy efficiency High pathway flux	High energy efficiency Low pathway flux	Low energy efficiency High pathway flux
Physiological and ecological implications	Fast growth and substrate conversion when plentiful resources available Improved conservation in energy‐limited conditions	Slow growth rates Symbiosis with others to acquire resources Adaption in oligotrophic environments	Integrated photosynthesis and respiration Main primary producer in oligotrophic waters

### 4.1 Chemoorganotrophs

Chemoorganotrophic bacteria acquire energy from organic molecules and typically prefer high‐flux rather than high‐efficiency energy pathways when their energy resources are not limited. For example, *E. coli* have no cytochrome *bc*_1_ complex in its ETC and dominantly use NDH‐2 under aerobic growth, which lacks proton translocation ability, rather than the proton‐pumping NDH‐1 for initial electron transport from NADH, leading to a total of only 2~4 protons translocated by ETC per molecule of NADH oxidised, corresponding to 0.6~1.2 ATP generation depending on which terminal oxidase (cytochrome *bo*_*3*_/*bd*‐I/*bd*‐II) is used (Puustinen et al. 1989, 1991; Calhoun and Gennis 1993; Unden and Bongaerts 1997; Borisov et al. 2008; Borisov, Murali, et al. 2011). In energy‐limited conditions (e.g., fumarate and DMSO respiration), however, the proton translocation capability of NDH‐1 becomes essential in *E. coli* , leading to four additional protons translocated per NADH oxidised and thus at most 2.4 ATP generation (Tran et al. 1997; Unden and Bongaerts 1997). While some organotrophs such as *Corynebacterium glutamicum* incorporate *cytochrome bc*_1_ in their ETCs, lacking expression of proton‐pumping NDH‐1 still results in an overall high‐flux, low‐efficiency strategy. The ETC in *C. glutamicum* is slightly more complex than that in *E. coli* ., with electrons initially transferred from NADH by NDH‐2 (or from other substrates such as succinate, lactate, and malate by alternative oxidoreductases) to menaquinone without proton translocation, followed by split branches, with one branch consisting of cytochromes *bc*_1_ and *aa*_3_ and the other consisting of a cytochrome *bd* terminal oxidase only, to translocate at most 6 protons per molecule of NADH, corresponding to a maximum ATP output of 1.5–2 per NADH (Bott and Niebisch 2003; Kabashima et al. 2009; Zelle et al. 2021).

### 4.2 Chemolithotrophs

Chemolithotrophs access energy from reduced inorganics (e.g., hydrogen, ammonia, hydrogen sulphide, ferrous iron) and typically possess ETCs with higher energy conservation efficiency than chemoorganotrophic bacteria. Among chemolithotrophs, one of the best studied group for ETCs is *Paracoccus* sp., in which electrons can be extracted from various inorganics including hydrogen and sulphur and then donated to the respiratory chain for energy conservation, likely through the cytochrome *c* pools (Friedrich 1997; Rother et al. 2001; Dambe et al. 2005). *P. denitrificans* has been widely used as a model organism for ETC studies. During aerobic respiration, one of its ETCs is identical to that of mitochondria, consisting of a proton‐pumping NADH dehydrogenase, ubiquinone, cytochrome *bc*_1_, and the terminal oxidase cytochrome *aa*_3_ (John and Whatley 1975). The NADH dehydrogenase (NDH‐1) has the electrogenic ratio of 2 H^+^/e^−^ as in *E. coli* . In addition to the mitochondria‐analogous pathway, *P. denitrificans* can utilise two other distinct pathways. In this case, electrons are transferred from the complex I to either of two branches, where one consists of a cytochrome *bb*_*3*_ and the other consists of cytochrome *bc*_1_ plus a non‐proton pumping cytochrome *c* oxidase (Van Spanning et al. 1995). Proton translocation in *P. denitrificans* is conferred by the NDH‐1, cytochrome *bc*_1_, cytochrome *aa*_3_, and cytochrome *bb*_3_. During typical aerobic growth, oxidation of NADH yields 5 H^+^/e^−^, i.e., 2 from NDH‐1, 2 from *bc*_1_, and 1 from *aa*_3_. Many other chemolithotrophs, such as the ammonia‐oxidising bacteria *Nitrosomonas europaea* and *Kuenenia stuttgartiensis*, also show a potential of high energy conservation efficiency by incorporating cytochrome *bc*_*1*_ complexes in their ETCs to improve the proton translocation and the PMF generation (Hooper and Nason 1965; Hollocher et al. 1982; Whittaker et al. 2000; Arp et al. 2002; Chain et al. 2003; Hedrich et al. 2011; Dibrova et al. 2013; Kartal et al. 2013; de Almeida et al. 2016).

### 4.3 Phototrophs

Cyanobacteria are ancient bacterial species capable of both photosynthesis and respiration, with these two processes integrated in the thylakoid membrane (Liu et al. 2012). The photosynthetic light reactions begin by splitting a water molecule in photosystem II (PSII), passing two electrons to plastoquinone (PQ). Reduced plastoquinol then diffuses through the thylakoid membrane and donates electrons to the cytochrome *b*_6_ *f* complex. The *b*_6_ *f* complex is commonly thought to facilitate a Q‐cycle, similar to the *bc*_1_ complex seen in previously discussed bacteria. However, because of its role in photosynthesis cyclic electron flow as well as the presence of heme *c*_n_ that is missing in the *bc*_1_ complex, it has been proposed that the *b*_6_ *f* complex may perform a ‘modified Q cycle’ instead (Cramer and Zhang 2006; Baniulis et al. 2008). Regardless of the mechanism, however, the proton translocation has been demonstrated to be consistent. The *b*_6_ *f* complex transfers one electron to either plastocyanin (PC) or cytochrome *c*_6_, where it can then be used to reduce photosystem I (PSI). The other electron is transferred via heme groups to reduce a new plastoquinone molecule as in the ‘low potential’ chain of the Q cycle. Within PSI, electrons are transferred via iron–sulphur clusters to ferredoxin, then to a ferredoxin‐NADP reductase, where NADPH is produced. Two electrons required to reduce one NADPH are produced from the splitting of one water molecule in PSII. The proton motive force is conferred by the water splitting, which releases two protons into the lumen, and cytochrome *b*_6_ *f*, which releases four. A total of six protons are therefore theoretically available for ATP synthase, which requires 4.67–5 protons to synthesise one ATP, resulting in approximately 1.2–1.28 ATP (Belkin et al. 1987; Kramer and Evans 2011).

The respiratory chain of cyanobacteria utilises many of the same components from the photosynthetic chain (Lea‐Smith et al. 2016). A number of oxidoreductases have been identified to accept electrons from NADH, NADPH, and succinate (Peschek et al. 2004). Only NDH‐1, analogous to that of *E. coli* , contributes to the proton motive force, while succinate dehydrogenase has been suggested to be the main contributor to reducing the PQ pool in the respiratory chain (Cooley and Vermaas 2001). Similarly, several terminal oxidases have been identified (Hart et al. 2005), which can either accept electrons directly from plastoquinol or from reduced PC or cytochrome *c*_6_ (Frazao et al. 1995), contributing to PMF generation.

## 5 Ecological Implication of Bacterial Respiratory Chains

The composition and organisation of bacterial respiratory chains vary widely among bacteria with different energy‐generation mechanisms. Even in heterotrophs that gain energy from organic carbon sources, respiratory chains can be diverse, as seen in *E. coli* and *C. glutamicum* . Chemolithotrophs, which derive energy from a vast array of inorganic sources, seem to incorporate a greater number of proteins and pathways into their electron transport chains, contributing to the flexibility of their metabolisms.

**FIGURE 2:**
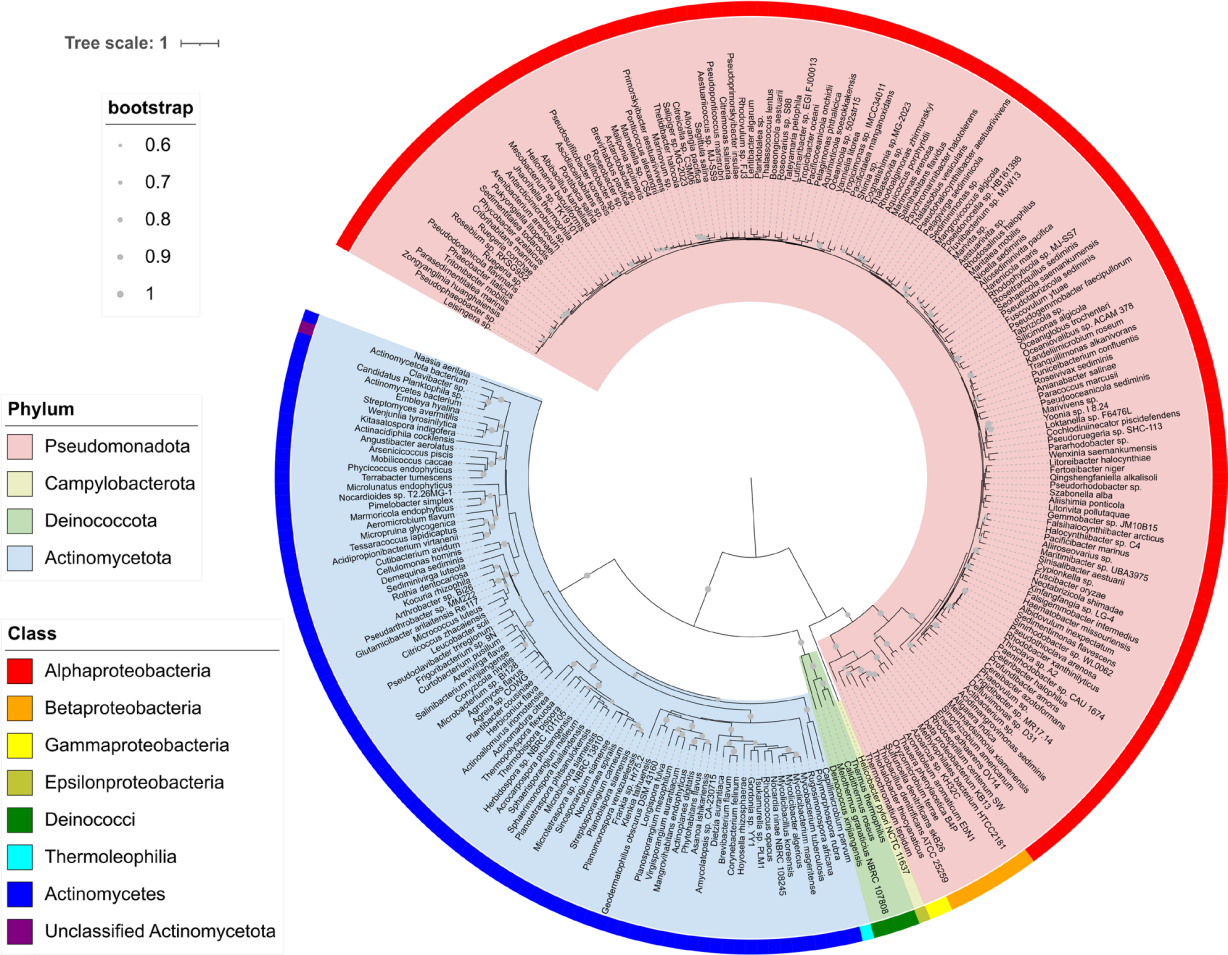
Diversity of the cytochrome b subunit of cytochrome *bc*_1_ complex in bacteria. One representative amino acid sequence for each genus (if available in databases) was included for the phylogenetic analysis. A maximum likelihood tree was constructed in MEGA11 using the best‐fit model and then annotated on the iTOL website to visualise the protein distribution across taxa. The coloured ranges (inner) and strips (outer) represent different phyla and classes respectively. The grey circles of varied sizes represent bootstrap values, with those below 0.6 not shown.

In general, bacteria that incorporate a *bc*_1_ complex, capable of using the Q‐cycle, have the capacity to generate higher PMF than those without it. We conducted a phylogenetic analysis using protein sequences of the cytochrome *b* subunit of cytochrome *bc*_1_ complex obtained from the NCBI protein database. While previous research using protein BLAST against genomes from the KEGG database predicted a border range of taxa potentially containing cytochrome *bc*_1_ complexes (Marreiros et al. 2016), we included only well‐annotated and available sequences to ensure a conserved analysis. The phylogenetics shows that cytochrome *bc*_1_ complexes are distributed among a wide variety of bacterial phyla, with most sequences in the database belonging to the alpha‐proteobacteria class and actinomycetota (actinobacteria) phylum (Figure 2). These two taxa represent the predominant bacteria in global oceans and soil, respectively (Sunagawa et al. 2015; Delgado‐Baquerizo et al. 2018; Chen et al. 2024). The metabolism of alpha‐proteobacteria is tremendously diverse, empowering them to survive in a variety of environments (Williams et al. 2007; Spain et al. 2009). Alpha‐proteobacteria include many chemolithotrophs such as ammonia‐oxidising bacteria, as well as other species such as *Acidithiobacillus ferrooxidans* that harvest energy from reduced ferrous iron or inorganic sulphur (Ingledew 1982). The employment of cytochrome *bc*_1_ complex in the ETC along with quinol and cytochrome *c* oxidases found in alpha‐proteobacteria (Degli Esposti et al. 2019) renders various species with different levels of energy conservation efficiencies and adaptability towards their energy needs.

**FIGURE 3:**
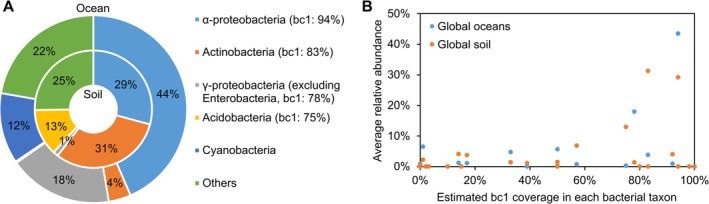
High prevalence of cytochrome *bc*_1_ complexes in dominant bacteria across global oceans and soil. (A) Taxonomic compositions of bacterial 16S rRNA gene sequences in global oceans (the outer ring) and soil (the inner ring). Major bacterial groups representing > 10% average relative abundance in either environment are shown. Values in parentheses indicate the estimated species coverage of predicted *bc*_1_ complexes within each respective taxonomic group. (B) Correlation between estimated *bc*_1_ complex coverage and average relative abundance across diverse bacterial taxa in global oceans (blue) and soil (orange). While many taxa across the spectrum of *bc*_1_ coverage remain at low relative abundance (< 5%), the most dominant bacterial groups (> 10% abundance) consistently exhibit high *bc*_1_ coverage (> 75%), suggesting that *bc*_1_ incorporation may represent a necessary metabolic adaptation for achieving high ecological abundance in global ocean and soil habitats.

To further explore the implication of cytochrome *bc*_1_ complexes in bacterial distribution, we retrieved the predicted *bc*_1_ complex species coverage data of different bacterial groups and their relative abundances in global oceans and soil from previous publications (Table S1) (Sunagawa et al. 2015; Delgado‐Baquerizo et al. 2018). Interestingly, we found that the most abundant taxa (excluding cyanobacteria), whose relative abundance exceeds 10%, consistently show a high occurrence level of cytochrome *bc*_1_ complexes (Figure 3A). When evaluating the relationship between average relative abundance and predicted *bc*_1_ complex coverage, the data indicate that high‐efficiency energy conservation may act as a prerequisite for ecological dominance. While many taxa with high *bc*_1_ coverage remain at low relative abundance (below 5%), the groups that achieve high population densities are almost exclusively those with high *bc*_1_ coverage exceeding 75% (Figure 3B). This observation suggests that while the presence of the *bc*_1_ complex does not guarantee ecological success, it provides the thermodynamic baseline required to support the metabolic demands of dominant bacterial populations in diverse habitats.

However, even with the proton‐translocating cytochrome *bc*_1_ complex, it seems that many bacteria do not fully exploit the energy potential in energy‐rich molecules such as NADH. This can be seen in many *Corynebacteria*, which use the non‐proton‐pumping NDH‐2 for NADH oxidation. During evolution, the flexibility in accessing different energy sources seems to take priority over energy efficiency. This might be advantageous for bacteria living in cycling habitats. For example, *E. coli* is most often found in the gastrointestinal tracts of humans and other mammals, but can also survive in so‐called secondary environments, like soil and water. Due to its need to cycle between host‐dependent and host‐independent environments, *E. coli* has developed a wide variety of mechanisms to adjust to different oxygenic and nutrient habitats. Some of these mechanisms include siderophore iron uptake and the use of ABC transporters for uptake of sugars and amino acids (van Elsas et al. 2011). The branched nature of the *E. coli* electron transport chain may thus play a role in its ability to adapt to such environmental niches.

Another consideration of energy metabolism is the trade‐off between rate and yield of ATP production. In the case of heterotrophs, fermentation pathways tend to have a high rate of ATP production while sacrificing the high yield produced by respiration. However, fermenters tend to outcompete respirators due to the high rate of substrate consumption. It has been suggested, however, that high ATP yield in respiration can lead to cooperation among cells to share resources (Pfeiffer et al. 2001), thus providing a selective advantage to multicellular organisms early in evolution. This inherent dilemma of ATP production rate versus yield may play an important role in the development of microbial metabolic diversity. Therefore, we propose the ecological significance of ETC energetics in driving microbial distribution and adaptation in environments. Further research into the evolution, diversity, and structure of these different electron transport chains could yield interesting insights into their relationship with their ecological niches. But one thing is certain, that nature has left little design space for us to further increase the energetic efficiency of the electron transport chain.

## Author Contributions

**Ningdong Xie:** conceptualisation, methodology, software, data curation, investigation, visualisation, writing – review and editing. **Allison M. Brown:** conceptualisation, investigation, writing – original draft. **Xin Wang:** conceptualisation, methodology, investigation, supervision, funding acquisition, project administration, writing – original draft, writing – review and editing.

## Funding

This work was supported by the National Science Foundation (2414925).

## Conflicts of Interest

The authors declare no conflicts of interest.

## Supporting information


**Table S1:** emi470338‐sup‐0001‐TableS1.xlsx. **Global distribution and relative abundance of bacterial taxa in relation to cytochrome *bc***_**1**_ **complex coverage**. This table summarises the predicted occurrence levels of the *bc*_1_ complex across various bacterial groups in global ocean and soil environments. Data integrates taxonomic relative abundance with *bc*_1_ genomic coverage to evaluate the role of high energy conservation as a potential prerequisite for ecological dominance. Sources: Sunagawa et al. (2015) for marine data and Delgado‐Baquerizo et al. (2018) for terrestrial data.

## Data Availability

Sequence data used for phylogenetic analyses were obtained from publicly available databases. The phylogenetic tree and compiled datasets generated in this study are provided in the main text and Supporting Information.
